# Pan-Genomic Analysis of *Clostridium botulinum* Group II (Non-Proteolytic *C. botulinum*) Associated with Foodborne Botulism and Isolated from the Environment

**DOI:** 10.3390/toxins12050306

**Published:** 2020-05-08

**Authors:** Jason Brunt, Arnoud H. M. van Vliet, Sandra C. Stringer, Andrew T. Carter, Miia Lindström, Michael W. Peck

**Affiliations:** 1 Department of Chemical Engineering and Biotechnology, University of Cambridge, Philippa Fawcett Drive, Cambridge CB3 0AS, UK; 2 Gut Health and Food Safety, Quadram Institute, Norwich Research Park, Norwich NR4 7UQ, UK; sandra.stringer@quadram.ac.uk (S.C.S.); andrewcarter594@btinternet.com (A.T.C.); 3 School of Veterinary Medicine, Faculty of Health and Medical Sciences, University of Surrey, Guildford GU2 7AL, UK; a.vanvliet@surrey.ac.uk; 4 Department of Food Hygiene and Environmental Health, Faculty of Veterinary Medicine, University of Helsinki, 00014 Helsinki, Finland; miia.lindstrom@helsinki.fi

**Keywords:** *Clostridium botulinum*, botulism, foodborne, neurotoxin, non-proteolytic

## Abstract

The neurotoxin formed by *Clostridium botulinum* Group II is a major cause of foodborne botulism, a deadly intoxication. This study aims to understand the genetic diversity and spread of *C. botulinum* Group II strains and their neurotoxin genes. A comparative genomic study has been conducted with 208 highly diverse *C. botulinum* Group II strains (180 newly sequenced strains isolated from 16 countries over 80 years, 28 sequences from Genbank). Strains possessed a single type B, E, or F neurotoxin gene or were closely related strains with no neurotoxin gene. Botulinum neurotoxin subtype variants (including novel variants) with a unique amino acid sequence were identified. Core genome single-nucleotide polymorphism (SNP) analysis identified two major lineages—one with type E strains, and the second dominated by subtype B4 strains with subtype F6 strains. This study revealed novel details of population structure/diversity and established relationships between whole-genome lineage, botulinum neurotoxin subtype variant, association with foodborne botulism, epidemiology, and geographical source. Additionally, the genome sequences represent a valuable resource for the research community (e.g., understanding evolution of *C. botulinum* and its neurotoxin genes, dissecting key aspects of *C. botulinum* Group II biology). This may contribute to improved risk assessments and the prevention of foodborne botulism.

## 1. Introduction

*Clostridium botulinum* is a polyphyletic taxon classified solely by the ability to form the deadly botulinum neurotoxin. It is separated into four groups (*C. botulinum* Groups I to IV) according to genotypic and phenotypic properties, and the four groups are sufficiently distinct as to be considered separate species [1,2,3,4,5,6,7,8]. The botulinum neurotoxin is also formed by some strains of *Clostridium baratii* and *Clostridium butyricum*, and a complete putative botulinum neurotoxin-encoding gene has been discovered in *Enterococcus*, with related putative genes in other non-clostridia [3,7,9,10,11,12]. Botulinum neurotoxins are the most potent toxins known and act in the cytoplasm of nerve cells, where they selectively cleave proteins involved in neurotransmitter release, resulting in a deadly flaccid paralysis called botulism [13,14,15]. For more than one hundred years, botulinum neurotoxins have been separated into serotypes using specific antisera to neutralise neurotoxin in animal tests [16,17]. More recently, sequencing of neurotoxin genes and the derivation of the amino acid sequence has led to the identification of subtypes within botulinum neurotoxin serotypes. A standardized numerical notation has been used for botulinum neurotoxin subtype nomenclature, and it has been proposed that a new subtype should diverge by at least 2.6% in amino acid sequence from an existing subtype [16]. More than 40 botulinum neurotoxin subtypes have been described, each comprising variants with a unique coding (and predicted amino acid) sequence. Interestingly, evidence is becoming available that botulinum neurotoxin subtypes display functional variation and possess distinct toxicological properties [18,19,20,21,22].

*C. botulinum* Group II isolates form a single type B, E, or F neurotoxin. Closely related non-toxic strains are also frequently described and are known as non-toxic (or non-neurotoxigenic) *C. botulinum* Group II. The botulinum neurotoxin is associated with accessory proteins in various complexes. Genes encoding the neurotoxin and accessory proteins are co-located in one of two conserved clusters (*ha* cluster or *orfX* cluster). Type B neurotoxin genes are located on a small multicopy plasmid (47–63 kb) in a *ha* cluster that comprises genes encoding the neurotoxin, non-toxic-non-haemagglutinin (NTNH), three haemagglutinins, and a positive regulator (*botR*). Type E and F neurotoxin genes are generally located on the chromosome (but for a small fraction of strains, the type E neurotoxin gene is present on a single-copy plasmid) in an *orfX* cluster that comprises genes encoding the neurotoxin, NTNH, and four open reading frames of unknown function [3,16,17,23,24,25,26,27,28,29,30,31].

Strains of *C. botulinum* Group II are widely distributed in the environment and may enter the food chain [32,33,34,35], potentially leading to foodborne botulism. Foodborne botulism is a severe and deadly intoxication caused by consuming food containing as little as 50 ng of botulinum neurotoxin [6,36]. *C. botulinum* Group II strains forming type B or type E neurotoxin are most frequently associated with foodborne botulism. Isolation of the first strain of *C. botulinum* was reported by Emile van Ermengem in 1897 following an outbreak of foodborne botulism involving salted ham in Belgium [37]. From the description of the bacterium, this was a strain of *C. botulinum* Group II that probably formed botulinum neurotoxin type B [16,17]. Unfortunately, this strain has been lost. 

*C. botulinum* Group II (also known as non-proteolytic *C. botulinum* or psychrotrophic *C. botulinum*) is a saccharolytic bacterium with a minimum growth temperature of 3 °C that forms spores of moderate heat resistance. Foodborne botulism outbreaks have been associated with temperature abuse of products intended to be stored chilled, including fish and meat, and continued extreme vigilance is necessary to ensure the safe production of mildly processed, vacuum/modified atmosphere-packed and other chilled foods [3,4,23,24,38,39,40,41]. 

Genomic analysis of *C. botulinum* Group II indicates that it can be separated into two (or three) major lineages depending on the strains tested and criterion applied [3,5,42,43,44,45,46,47,48,49]. The majority of strains forming type E neurotoxin are located in one lineage that is distantly separated from the other lineage that is dominated by strains forming subtype B4 neurotoxin and contains strains forming subtype F6 neurotoxin. Strains forming type E neurotoxin are frequently isolated from fish and the arctic/subarctic environment, but have now also been found in the southern hemisphere [6,50,51]. Strains forming subtype B4 neurotoxin have been previously isolated from European terrestrial environments (including pigs) and from marine (notably, north America) environments [23,51]. 

At the start of the present study, there were only 28 genomes available in Genbank for *C. botulinum* Group II. The purpose of the present study is to provide insights into the genetic diversity and spread of strains of *C. botulinum* Group II and their neurotoxin genes. To achieve this, the genomes of 180 additional, highly diverse strains of *C. botulinum* Group II were sequenced. The 208 strains studied were isolated from 16 countries and four continents over a period of over 80 years. This is the most comprehensive comparative genomic study of *C. botulinum* Group II undertaken to date. Additionally, the total of 208 genome sequences represent a valuable resource for the research community—for example, contributing to understanding the evolution of *C. botulinum* Group II and its neurotoxin genes, to the dissection of key aspects of the biology of *C. botulinum* Group II important for food chain transmission, such as spore formation, spore germination [52,53], psychrotrophy [54], neurotoxin formation [55,56,57], and in the targeting of PCR assays to detect and distinguish strains. Such information will contribute to more comprehensive microbiological risk assessments, the safe development of novel “fresh-like” foods, and the prevention of foodborne botulism.

## 2. Results and Discussion

### 2.1. Botulinum Neurotoxins and Their Encoding Genes

**Figure 1:**
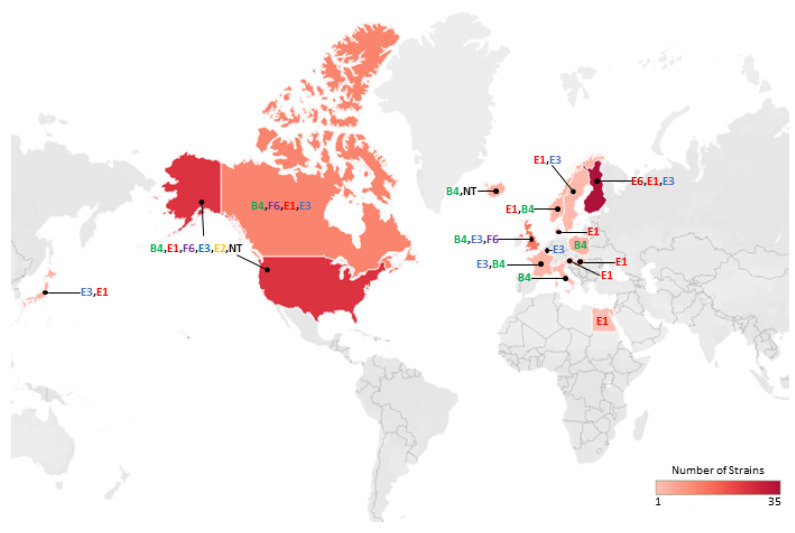
Geographical locations and heat map of newly sequenced isolates used in this study. A total of 113 newly sequenced isolates were attributed to a geographical location. Further details about individual isolates are given in Table S1.

This study analysed the genomes of 208 strains of *C. botulinum* Group II. This comprised 180 newly sequenced genomes, and 28 genomes from public sources. The newly sequenced genomes considerably increase the number of genome sequences publicly available for *C. botulinum* Group II. The sequenced strains were from a wide geographical area (16 countries over four continents; Figure 1), with the first strain isolated in 1934. Thirty-seven strains were associated with foodborne botulism outbreaks, 94 strains were non-clinical isolates not associated with foodborne botulism outbreaks (i.e., isolated from the environment), and the status of the remaining strains is unknown (Table S1). The strains possessed a single neurotoxin gene that encoded neurotoxin of subtype B4, E1, E2, E3, E6 or F6 or there was no neurotoxin gene. No genes were identified that might encode previously undescribed botulinum neurotoxin serotypes or subtypes. Variants, with a unique coding sequence (and predicted amino acid sequence), were identified for each neurotoxin subtype. 

**Figure 2:**
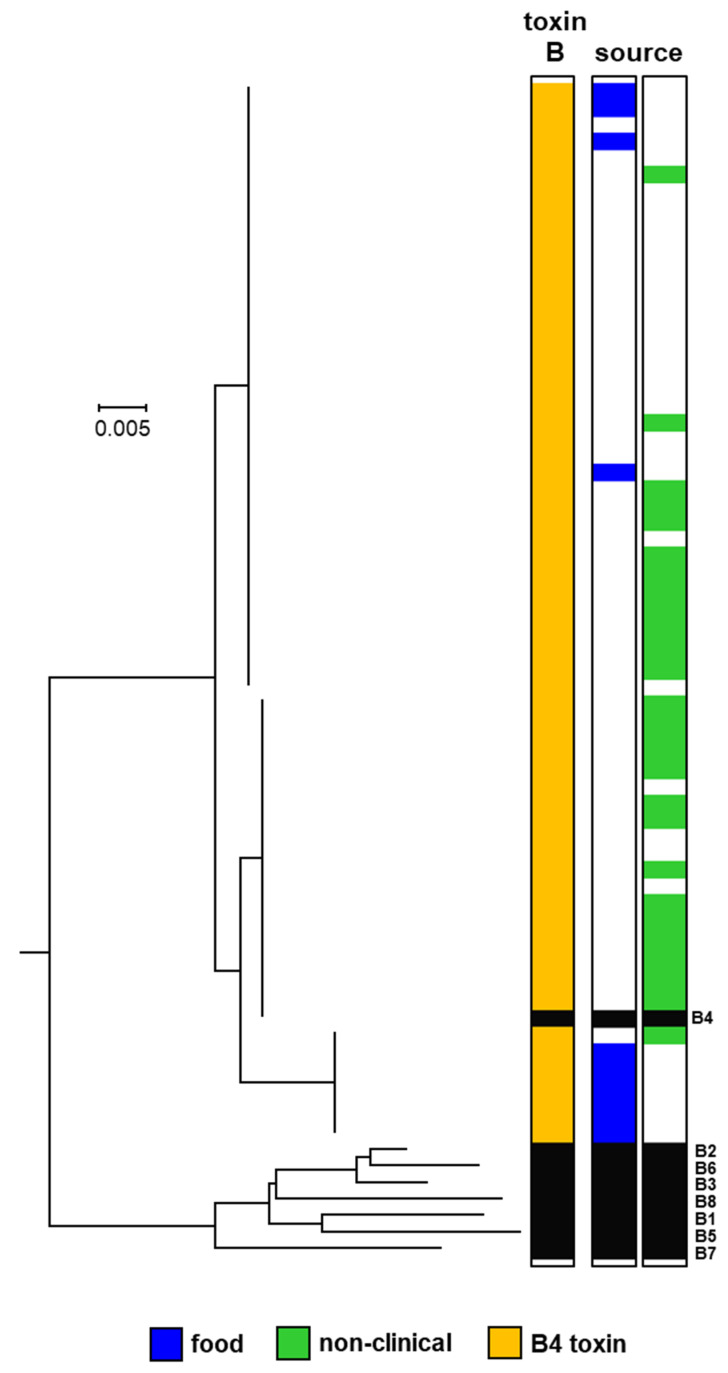
Phylogeny of subtypes of botulinum neurotoxin type B. Further details about individual sequences and isolates are given in Table S2, where they appear in the same order as in this figure. The sources of the isolates are given in Table S1. The toxin protein sequences were aligned with the Muscle module of MEGA7 [59] algorithm and the phylogenetic tree was generated using the Neighbour-Joining method. Three subtype B4 neurotoxin variants are present in *C. botulinum* Group II. Scale bar represents the number of amino acid substitutions per site. A total of 71 amino acid sequences were analysed. White blocks represent absence of information regarding source. Black blocks represent reference neurotoxins [16].

Eight subtypes of type B neurotoxin (B1 to B8) have been described that differ by a maximum of 7.1% amino acid residues [15]. Botulinum neurotoxin subtype B4 is exclusively formed by strains of *C. botulinum* Group II, and a previous survey of 16 examples of this neurotoxin revealed amino acid differences of 1.6% [3,16]. The genome of 63 *C. botulinum* Group II strains examined in the present study contained a subtype B4 neurotoxin gene within a typical *ha* neurotoxin gene cluster arrangement. No other genes encoding entire or partial botulinum neurotoxins were detected in these genomes. From analysis of the B4 botulinum neurotoxin-encoding genes, three distinct variants of this subtype were identified (Figure 2). These three variants have been previously described for a smaller number of strains [3,5,16,23], and it is notable that no new variants were identified in this larger study, suggesting that future subtype B4 neurotoxins may also fall within the same arrangement. Although information is not available for all strains, there is evidence of potential epidemiological and geographic distribution bias. The largest number of strains were in the upper subtype B4 variant group (Figure 2). Where known, these strains were all isolated in Europe, and either associated with foodborne botulism or were isolated from the environment (non-clinical isolates). The foodborne botulism outbreaks involved the consumption of meat in Iceland, Italy and the U.K. [23,58]. The U.K. outbreak was associated with the consumption of home-prepared pork that had been home slaughtered, bottled and kept at ambient temperature for several months in Poland before being brought to the U.K. Following treatment with botulinum antitoxin, the patient made a full recovery [58]. A single subtype B4 variant was recently found in all seven strains isolated in France and associated with six foodborne botulism outbreaks involving meat [43]. All strains in the centre subtype B4 variant group (Figure 2) were from the environment (non-clinical isolates) and were principally from north America. The seven strains in the lower subtype B4 variant group were all isolated in north America, six following outbreaks of foodborne botulism involving fish, and the seventh from Pacific Ocean sediments (Figure 2). 

There are currently 12 recognised subtypes of botulinum neurotoxin type E (E1 to E12). These subtypes are generally closely related (amino acid difference of 0.9–5.9%), although subtypes E9 and E12 are more diverse [16]. The genomes of 98 strains contained a type E neurotoxin gene, all belonging to known subtypes [16]. The genome of 58 strains contained a subtype E1 botulinum neurotoxin gene, three strains contained a subtype E2 botulinum neurotoxin gene, 30 strains a subtype E3 gene, and seven strains a subtype E6 gene. This is a valuable addition to the existing sequence data that was heavily focused on subtype E3 [16]. All type E neurotoxin genes were located within a typical *orfX* neurotoxin gene cluster. 

**Figure 3:**
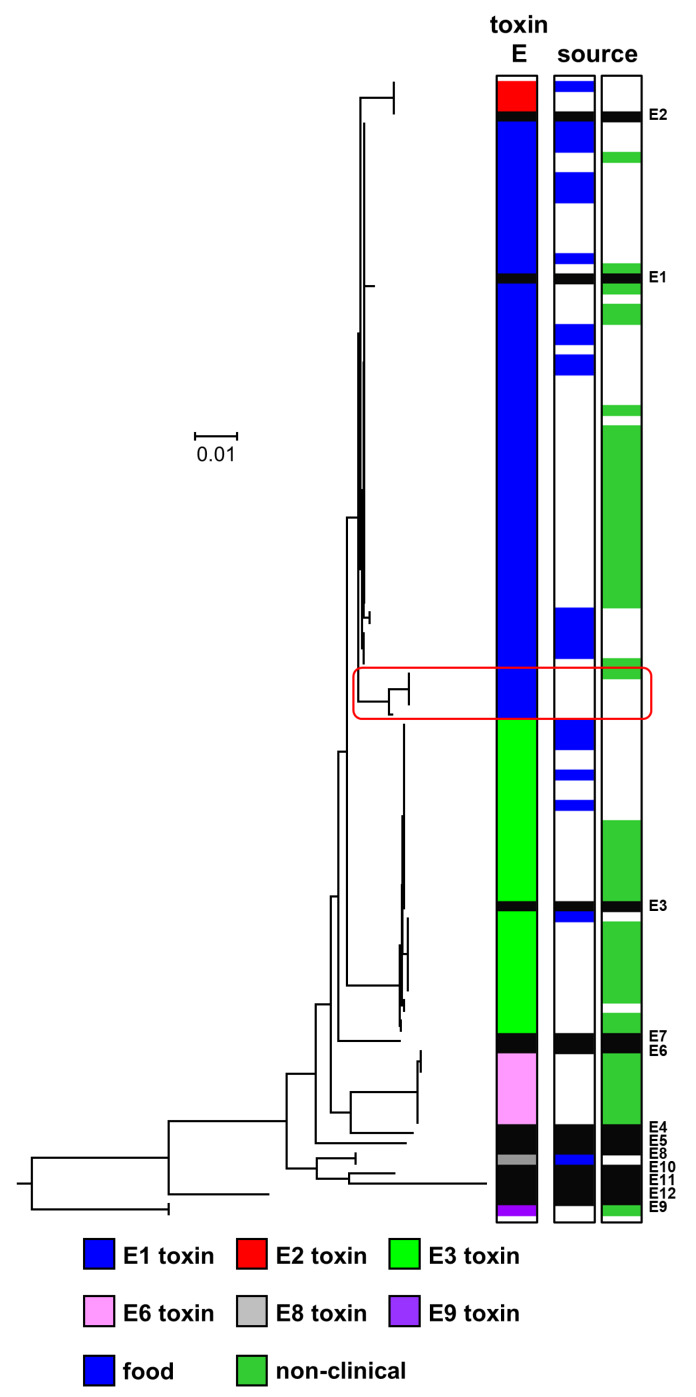
Phylogeny of subtypes of botulinum neurotoxin type E. Further details about individual sequences and isolates are given in Table S2, where they appear in the same order as in this figure. The sources of the isolates are given in Table S1. The toxin protein sequences were aligned with the Muscle module of MEGA7 [59] algorithm and the phylogenetic tree was generated using the Neighbour-Joining method. The new variants of subtype E1 are highlighted in a red box. Scale bar represents the number of amino acid substitutions per site. A total of 112 amino acid sequences were analysed. White blocks represent absence of information regarding source. Black blocks represent reference neurotoxins [16].

Fifty-eight strains possessed a gene encoding subtype E1 botulinum neurotoxin (Figure 3). These strains appear widespread, and the strains examined in the present study have been isolated over a period of more than 70 years from northern Europe (Finland, Sweden, Denmark and Norway), central Europe (Austria and Hungary), north America (Canada and USA), Africa (Egypt), and Asia (Japan), with many associated with foodborne botulism (Table S1). Several variants of subtype E1 botulinum neurotoxin were identified (Figure 3), as noted previously [16,42]. The sequence for subtype E1 neurotoxin derived from whole-genome sequences for two isolates of strain Beluga from our collection (CB0187, CB0603) and one from the USA (CB1085) were identical to each other and also that derived from whole-genome sequences of other strains (Figure 3), but different on eight positions out of 1252 to the E1 toxin in accession number CAA43999, which is commonly used as the standard E1 toxin. In future comparisons, there may be merit in using the subtype E1 neurotoxin sequence in genome accession ACSC01 (CB1085) as the new standard. Interestingly, the present study revealed five amino acid sequences (strains Saroma, SAR, 1304E, IFR 18/112, IFR 18/132) that form two new closely related variants of subtype E1 (highlighted in red box in Figure 3). Two of the strains (Saroma and 1304E) are non-clinical isolates and were probably isolated from lake sludge in Hokkaido Island, Japan, before 1970. Additionally, “SAR” may be an abbreviation for Saroma, indicating a similar origin. Amino acid alignment revealed that four of these new subtype E1 sequences (strains; Saroma, SAR, IFR 18/112, IFR 18/132) shared 98.6% identity with the subtype E1 toxin sequence in genome accession ACSC01 (CB1085), while the toxin of strain 1304E shared 99.0% identity with subtype E1 toxin sequence at the amino acid level (and they also shared 97.9% and 98.3% identity with the E2 neurotoxin, respectively). Guidelines indicate that a 2.6% amino acid difference should be used to separate botulinum neurotoxin subtypes [16]. However, although botulinum neurotoxin subtypes E1, E2 and E3 are distinct, the amino acid differences between these subtypes is less than 2.6%. Thus, if the 2.6% amino acid difference guideline to separate botulinum neurotoxin subtypes was applied, then these would be considered a single subtype [16]. The new subtype E1 variant sequences described here are 1.0% to 1.4% different from subtype E1, and therefore do not meet the above-described criterion for a new botulinum neurotoxin subtype and have therefore been designated as subtype E1. Botulinum neurotoxins consist of two chains, forming an N-terminal light chain (LC) and a C-terminal heavy chain (HC) which are linked by a disulphide bond. The HC is additionally separated into an N-terminal translocation domain (H_N_) and a C-terminal receptor-binding domain (H_C_). Further analysis of the novel subtype E1 variants amino acid sequence revealed non-conservative amino acid replacements in the translocation domain (Figure S1). Whether these changes affect neurotoxin translocation into the host cytosol remains unknown.

Three strains possessed an identical gene encoding subtype E2 botulinum neurotoxin (Figure 3). Two of these strains were isolated following foodborne botulism outbreaks in Alaska [42,45]. The source of the third strain is not known. Thirty strains possessed a gene encoding subtype E3 botulinum neurotoxin (Figure 3). The strains were from diverse geographical locations, including various countries over three continents (Europe, north America and Asia), and were associated with foodborne botulism or isolated from the environment (non-clinical isolates) or from an unknown source (Table S1). Four variants of the subtype E3 botulinum neurotoxin were identified (Figure 3). MacDonald et al. [42] previously found three subtype E3 botulinum neurotoxin variants amongst 16 strains, while Weedmark et al. [60] identified nine subtype E3 variants within 125 subtype E3 strains isolated from widely distributed sites throughout much of northern Canada. The same subtype E3 variant (present in strain Alaska E43) was dominant in both the present and previous studies [42,60]. The seven strains that possessed a gene encoding subtype E6 botulinum neurotoxin were all non-clinical isolates from fresh fish, fish product, or fishpond sediments from different geographical locations in Finland (Table S1). Two variants of the subtype E6 botulinum neurotoxin were identified (Figure 3). Interestingly, none of 175 *C. botulinum* Group II type E strains associated with foodborne botulism or isolated from the environment (non-clinical isolates) in northern Canada possessed a subtype E6 botulinum neurotoxin gene [60].

**Figure 4:**
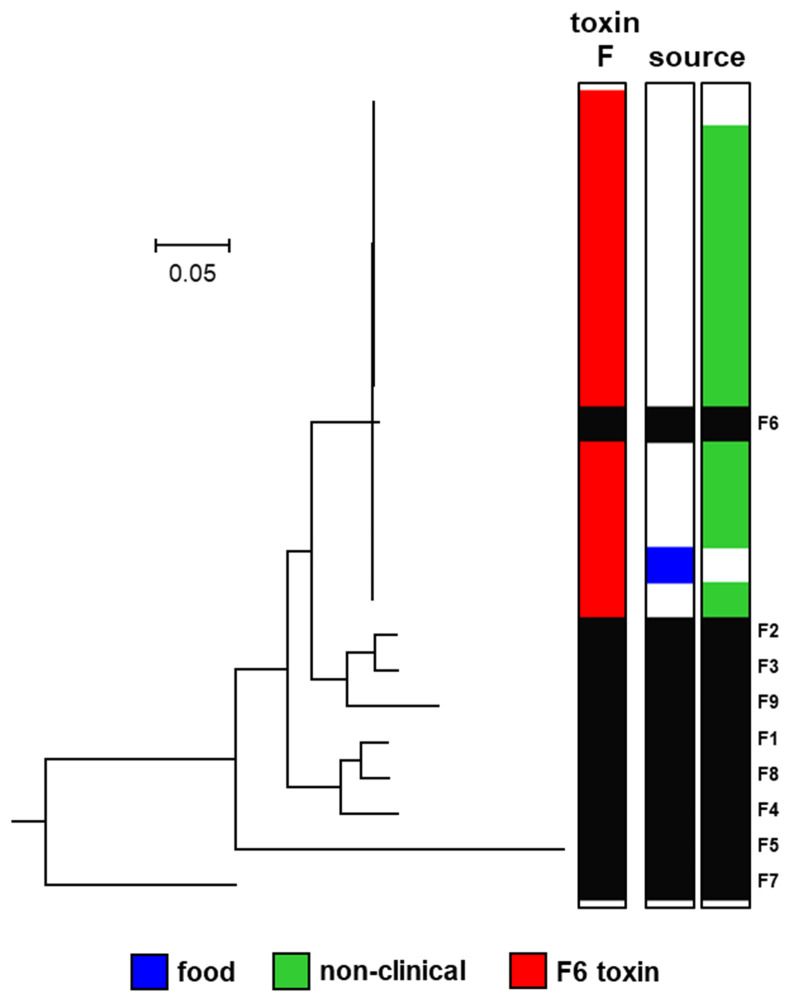
Phylogeny of subtypes of botulinum neurotoxin type F. Further details about individual sequences and isolates are given in Table S2, where they appear in the same order as in this figure. The sources of the isolates are specified in Table S1. The toxin protein sequences were aligned with the Muscle module of MEGA7 [59] algorithm and the phylogenetic tree was generated using the Neighbour-Joining method. Scale bar represents the number of amino acid substitutions per site. A total of 23 amino acid sequences were analysed. White blocks represent absence of information regarding source. Black blocks represent reference neurotoxins [16].

Botulinum neurotoxin type F is highly diverse. Eight subtypes (F1 to F8) are recognised that differ by up to 36.2% of amino acid residues [15]. Botulinum neurotoxin subtype F6 is exclusively formed by strains of *C. botulinum* Group II, and small differences (amino acid difference of 0.2%) were described between seven examples of this botulinum neurotoxin subtype [3,16]. The 14 strains of *C. botulinum* Group II type F examined in the present study comprised one isolate from an outbreak of foodborne botulism involving venison jerky in USA [61], with most of the remaining strains being non-clinical isolates [25], and one strain unknown. A majority of the strains originated from north America (Table S1). All strains possessed a gene encoding subtype F6 botulinum neurotoxin (Figure 4). Previous studies have reported the subtype F6 neurotoxin gene to be the only type F neurotoxin gene present in *C. botulinum* Group II [3,16]. Two variants of the subtype F6 neurotoxin gene were identified, with the encoded neurotoxins >99% identical. All subtype F6 genes were located within a typical *orfX* neurotoxin gene cluster. Remnants of type B and type E botulinum neurotoxin genes were also detected in all *C. botulinum* Group II type F strains, as described previously [25].

### 2.2. Whole-Genome Analysis Based on Single-Nucleotide Polymorphisms (SNPs)

**Figure 5:**
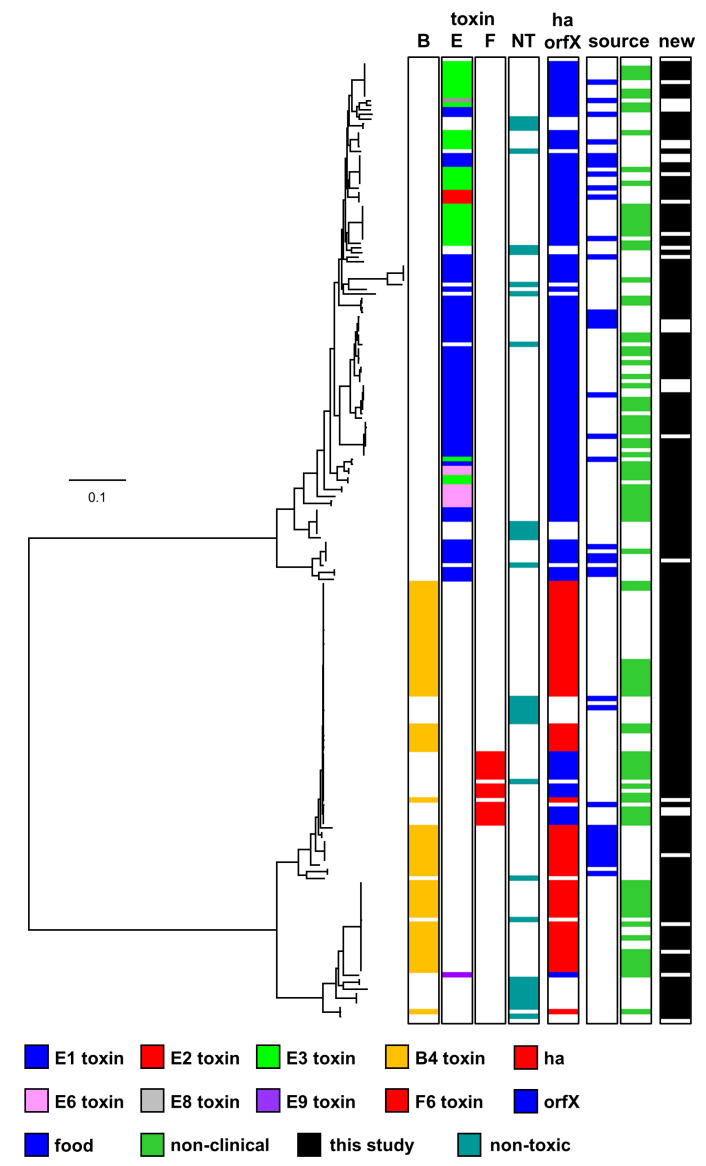
Phylogeny of *C. botulinum* Group II genomes. Two major lineages (type E neurotoxin gene lineage and type B/E/F neurotoxin gene lineage) were identified. The phylogenetic tree was created by comparison of core single-nucleotide polymorphisms identified using the ParSNP program [63]. Treegraph v2 [64], MEGA7 [59] and Figtree were used to annotate and visualize the phylogenetic tree. Accessory genes were identified using BLAST, using reference sequences for the *ha* and *orfX* neurotoxin cluster configurations. The distance bar (0.1) represents the number of nucleotide substitutions per site for a given branch, based on the number of SNPs found in the core genome. Further details about individual sequences and strains are given in Tables S1 and S3, where they appear in the same order as in this figure.

*C. botulinum* Group II is reported to be a more diverse bacterium than *C. botulinum* Group I, with less horizontal gene transfer and fewer recombination events [1,3,6,8,48,49]. Various techniques including comparison of core genome single-nucleotide polymorphisms (SNPs) following whole-genome sequencing and comparative genomic indexing (using a DNA microarray) have been employed to understand the diversity and population structure, and the evolutionary and phylogenetic relationship between strains of *C. botulinum* Group II [2,5,6,8,42,43,44,45,46,62]. In the present study, the genomic diversity of 208 strains of *C. botulinum* Group II has been investigated based on an analysis of core genome SNPs following whole-genome sequencing (Figure 5). Two major lineages have been identified. The first lineage contains strains possessing a type E neurotoxin gene (referred to as type E toxin gene lineage) and is distantly separated from the other lineage. The other lineage (referred to as type B/E/F toxin gene lineage) separates into two closely related sub-lineages, dominated by strains possessing a subtype B4 toxin gene, and also includes strains possessing a subtype F6 toxin gene and a small number of strains with a type E toxin gene (Figure 5). Strains lacking a botulinum neurotoxin gene were present throughout both lineages (Figure 5) and closely related to strains possessing a neurotoxin gene on a plasmid or the chromosome [3,23,24,25,26,27,28]. Type E and subtype F6 genes were located within an *orfX* neurotoxin gene cluster, while subtype B4 genes were located within a *ha* neurotoxin gene cluster (Figure 5). It is notable that the population and diversity structure identified in the present study contains no additional major lineages, and is consistent with that made in previous studies that have largely been carried out on a smaller number of and/or less diverse strains [2,5,6,8,42,43,44,45,46,49,62]. This suggests that further strains of *C. botulinum* Group II may keep to the same arrangement.

A comparison of the physiological response of strains within these two lineages has revealed a relationship between lineage (and toxin serotype) and carbohydrate utilisation pattern, but not growth response at chill temperature or at high NaCl concentrations [45]. However, a systematic assessment of literature data has established a relationship between lineage (and toxin serotype) and spore heat resistance, despite methodological differences amongst the many studies reviewed [65]. A detailed analysis of the difference in physiological response of strains in these lineages, as well as a genome-wide association study to identify the genetic mechanisms behind each physiological response, is merited. Such information could make a valuable contribution to future microbiological risk assessments [44]. In challenge test experiments where spores from a mixture of strains of *C. botulinum* Group II are added to a food and the potential for neurotoxin formation assessed, it would seem prudent to include strains from both lineages.

The upper lineage shown in Figure 5 includes the majority of strains with genes encoding type E neurotoxin—notably, strains with genes encoding botulinum neurotoxin subtypes E1, E2, E3, E6 and E8, and closely related strains lacking a neurotoxin gene. No strain possessed additional neurotoxin-encoding genes. This is consistent with previous studies [42,44,45,46]. It has been proposed that these strains and their type E neurotoxin genes arose from the insertion of the type E neurotoxin gene into genetically conserved bacteria, followed by recombination events [42]. Strains possessing a gene encoding subtype E1 neurotoxin appeared more diverse (based on core genome SNPs analysis), and were isolated over a longer period of time and wider geographical location (northern Europe, central Europe, north America, Africa, and Asia) than strains possessing genes encoding subtypes E2, E3 or E6 neurotoxin (Table S1), although it should be noted that more strains possessing a gene encoding subtype E1 neurotoxin were included in the present study. Strains throughout this lineage were associated with foodborne botulism or isolated from the environment (non-clinical isolates) or from an unknown source (Figure 5). Foodborne botulism was generally associated with the consumption of fish.

The two lower sub-lineages shown in Figure 5 are both dominated by strains possessing a subtype B4 toxin gene and are more closely related to each other than to the upper type E lineage. The uppermost sub-lineage contains strains with a gene encoding subtype B4 or subtype F6 neurotoxin and also closely related strains lacking a neurotoxin gene (Figure 5). Strains with a gene encoding subtype B4 neurotoxin possessed no additional neurotoxin-encoding genes, while strains with a gene encoding subtype F6 neurotoxin possessed remnants of other botulinum neurotoxin genes, as noted previously [25]. The strains with a gene encoding subtype F6 neurotoxin are very closely related and are proposed to have arisen once following a series of events that included the insertion of a 34 kb cassette that includes a gene encoding subtype F6 neurotoxin into an ancestral type B strain, and inactivation of the type B gene [25]. Interestingly, strain DB-2, which was collected from Pacific Ocean sediments [23], possessed a subtype B4 neurotoxin gene but is very closely related to strains with a gene encoding subtype F6 neurotoxin (Figure 5). This confirms previous observations based on whole-genome analysis that strain DB-2 is more closely related to *C. botulinum* Group II type F strains 202F and 610F than to other *C. botulinum* Group II type B strains [43,46]. Strains throughout this sub-lineage were associated with foodborne botulism or isolated from the environment (non-clinical isolates) or an unknown source. Foodborne botulism was associated with the consumption of meat or fish contaminated with botulinum neurotoxin in Europe or north America. Although not included in the present study, it is likely (based on whole-genome analysis) that a subtype E12 strain (84–10) isolated in France also locates to this sub-lineage [46]. Thus, this sub-lineage is likely to include *C. botulinum* Group II type B, E, and F strains.

The lower sub-lineage shown in Figure 5 contains strains possessing a subtype B4 toxin gene, a type E toxin gene, and also closely related strains lacking a neurotoxin gene. Strains possessed no more than one neurotoxin-encoding gene. Previous reports indicate that only a small number of strains possessing a type E toxin gene are located in this sub-lineage [27,43,44,46,50,66]. Two of the isolates present within the lower sub-lineage (Figure 5) and lacking a botulinum neurotoxin gene were derived in our laboratory from strains that possessed a subtype B4 neurotoxin gene. One of these isolates was characterized in some detail. Except for an inability to form botulinum neurotoxin, no physiological differences were identified between the non-toxic isolate (BL81/22NT, CB0395) and the original toxic strain (Eklund 17B(3), CB0597). However, the toxic strain possessed the entire neurotoxin-encoding plasmid p17BNRP, which was completely absent from the non-toxic isolate. None of the strains examined in the present study in the lower sub-lineage were associated with foodborne botulism.

It is important to differentiate *C. botulinum* Groups and indeed their lineages, especially when investigating outbreaks of botulism involving humans or animals. Traditionally, identification relied on growing cultures that would be subsequently tested using either mouse bioassays, ELISAs, mass spectrometry, toxin activity assays, and/or PCR assays for toxin gene presence. One major advantage of the PCR assay is the speed of the test. Williamson and colleagues [47] have developed an in silico multiplex PCR assay to differentiate two major distinct lineages of *C. botulinum* Group II (i.e., type E toxin gene lineage, and type B/E/F toxin gene lineage; Figure 5). The sequencing work in the present study has greatly increased the number of genomes now publicly available, and we have evaluated whether these new additional genomes were correctly assigned using an established in silico multiplex PCR [47]. Of the 208 strains tested in the present study, the in silico PCR assigned 207 strains into the correct major lineage of *C. botulinum* Group II. Only strain IFR 18/139 (CB0543) was not correctly assigned. Upon further analysis, the in silico PCR failed to identify this strain partially due to the target sequence being split over two contigs and additional rare mutations. In reality, conventional PCR would feasibly detect strain IFR 18/139 as in silico PCR is more stringent and requires a complete sequence match. The findings therefore support the continued use of this in silico multiplex PCR [47].

### 2.3. Pan-genome Comparison of the Two C. botulinum Group II Genomic Lineages

**Figure 6:**
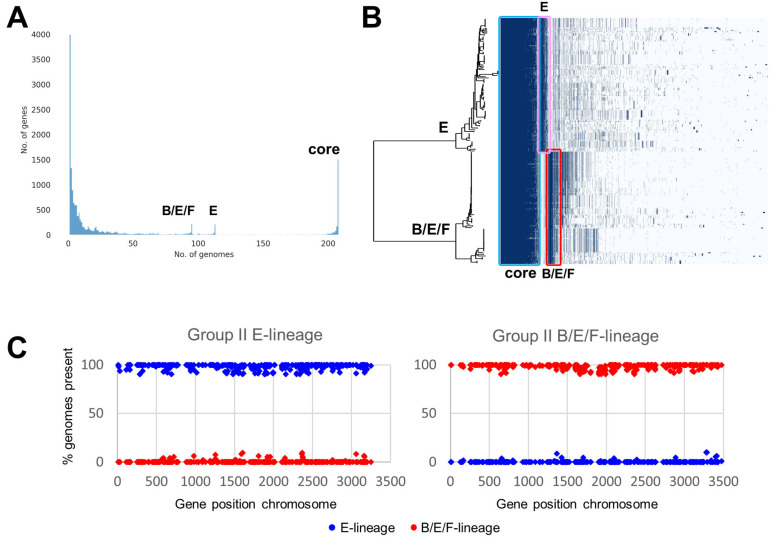
Pan-genome comparison of the two major lineages of *C. botulinum* Group II. The two major lineages (type E neurotoxin gene lineage and type B/E/F neurotoxin gene lineage) are shown in Figure 5. (**A**) Distribution of features per genome, with many features only present in a few genomes. The core features are shown at the right-hand side, and the type E toxin gene lineage and type B/E/F toxin gene lineage-specific genes are found in the centre. (**B**) Graphical representation of feature distribution linked to the SNP-based phylogenetic tree also used in Figure 5. The features marked by the blue square represent the core genome, the pink square represents the genes specific for the type E toxin gene lineage, and the red square represents the genes specific for the type B/E/F toxin gene lineage. (**C**) The lineage-specific genes are distributed throughout the genome, as shown using representative genomes for the type E toxin gene lineage (Alaska E43) and type B/E/F toxin gene lineage (Eklund 17B-NRP). Dark blue diamonds represent type E toxin gene lineage features, and red diamonds represent type B/E/F toxin lineage features.

The subdivision of the *C. botulinum* Group II genomes into two distinct genomic lineages, one containing 113 genomes with different subtypes of the type E toxin gene (type E toxin gene lineage), and one containing 95 genomes primarily with subtype B4 toxin gene or subtype F6 toxin gene or more rarely type E toxin gene (type B/E/F toxin gene lineage) (Figure 5) was used to search for lineage-specific genes. The pangenome of *C. botulinum* Group II was generated using Prokka-annotated genome sequences and the Roary software package [67], followed by identification of lineage-specific genes defined as present in ≥90% of genomes of the lineage, and ≤10% of genomes in the other lineage. The *C. botulinum* Group II pan-genome contained a total of 16,571 annotated features (Figure 6A), with a core genome of 1768 features present in >99% of all genomes from both lineages. There were 390 features specific for the type E toxin gene lineage, and 409 features specific for the type B/E/F toxin gene lineage (Figure 6B). Two complete genomes were selected as representatives for each lineage, Alaska E43 for the type E toxin gene lineage, and Eklund 17B-NRP for the type B/E/F toxin gene lineage, and the relative position of genes specific for their lineage were plotted (Figure 6C). The lineage-specific genes were spread throughout the chromosome, without a clear hotspot for integration such as mobile elements, plasmids or phages. While a detailed investigation of the lineage-specific genes falls outside the scope of this study, we note that the type E toxin gene lineage contains a histidine biosynthetic pathway (*hisSZGDCBHAFIEJ*) absent from the type B/E/F toxin gene lineage, indicating that histidine metabolism or potential auxotrophy may be used to distinguish these two lineages. Conversely, no comparable features were identified in the type B/E/F toxin gene lineage, and hence this may require more in-depth investigation.

## 3. Conclusions

This comparative genomic study of 208 highly diverse strains of *C. botulinum* Group II (180 newly sequenced strains, 28 sequences from Genbank) has considerably increased the number of available genomes for this bacterium. New findings have been made with respect to population structure, diversity and spread, and novel relationships have been established between whole-genome lineage, botulinum neurotoxin subtype variant, association with foodborne botulism, epidemiology, and geographical source. Strains possessed a single type B, E, or F neurotoxin gene. No genes were identified that might encode previously undescribed botulinum neurotoxin serotypes or subtypes, potentially indicating that there are few presently undescribed neurotoxin serotypes or subtypes associated with *C. botulinum* Group II. However, novel botulinum neurotoxin subtype variants were identified, each with a unique amino acid sequence. Core genome SNPs analysis revealed two major lineages—one with type E strains (type E toxin gene lineage), and the second with two closely related sub-lineages dominated by subtype B4 strains that also included subtype F6 strains and a small number of type E strains (type B/E/F toxin gene lineage). Both lineages contained closely related strains that lacked a botulinum neurotoxin-encoding gene. The absence of new lineages and the finding that the population diversity organisation is similar to that described previously based on fewer and/or less diverse strains suggest that further strains of *C. botulinum* Group II may also follow the same organisation. The *C. botulinum* Group II pan-genome contained 16,571 annotated features, with a core genome of 1768 features present in >99% of all genomes, 390 features specific to the type E toxin gene lineage, and 490 features specific to the type B/E/F toxin gene lineage. The lineage-specific genes were spread throughout the chromosome, with no integration hotspot. Finally, the genome sequences are a valuable resource—for example, contributing to future research on the evolution of *C. botulinum* Group II and its neurotoxin genes, the dissection of key aspects of the biology of *C. botulinum* Group II essential for food chain transmission, and the targeting of PCR assays to detect and distinguish strains. This may contribute to improved microbiological risk assessments, the safe development of novel “fresh-like” foods and the prevention of foodborne botulism.

## 4. Materials and Methods

### 4.1. C. botulinum Group II Isolates

The genomes of 208 isolates were assigned to *C. botulinum* Group II based on their genome sequence. This comprised 180 newly sequenced genomes, and 28 genomes from public sources. The newly sequenced genomes either possessed a single neurotoxin gene of subtype B4 (63 strains), E1 (58 strains), E2 (3 strains), E3 (30 strains), E6 (7 strains), F6 (14 strains) or no neurotoxin gene (33 strains). Strains with a complete subtype F6 gene also contained fragments of other neurotoxin genes. It was not determined whether the presence of a neurotoxin gene was associated with formation of botulinum neurotoxin. The newly sequenced strains were from 16 countries and four continents (Figure 1); with the first strain isolated in 1934. Thirty-seven strains were associated with outbreaks of foodborne botulism, 94 strains were non-clinical isolates not associated with foodborne botulism outbreaks (i.e., isolated from the environment), and the status of the remaining strains is unknown (Table S1).

### 4.2. Genomic DNA Preparation

DNA extraction was performed by following the traditional Gram-positive cell lysis procedures. Briefly, cell pellets from an overnight 10 mL TPYG broth culture (30 °C) were treated with lysozyme, mutanolysin and RNase A in TE buffer at 37 °C, 60 min, followed by lysis in proteinase K, SDS and NaCl for a further 60 min at 55 °C. The clear lysate was purified by phenol-chloroform, then chloroform/IAA extraction before ethanol precipitation and dissolution in TE buffer [68].

### 4.3. Whole-Genome Sequencing

Whole-genome sequencing of the *C. botulinum* gDNA samples was carried out at the Earlham Institute, Norwich, UK. Libraries were constructed using 1 ng of input DNA in their low input transposase-enabled (LITE) low-cost, high-throughput library construction pipeline based on the Illumina Nextera kits (Illumina, Cambridge, UK). Each library was constructed using unique 9 bp dual index combinations allowing samples to be multiplexed. Pooled libraries were then sequenced with a 2 × 250 bp read metric on an Illumina HiSeq 2500 sequencer.

### 4.4. Genome Assembly and Quality Control

Genomes were assembled using Shovill version 1.0.4 (https://github.com/tseemann/shovill), using the Spades 3.13.0 assembler module [69] and Trimmomatic [70] enabled. Contigs < 200 bp and coverage <5-fold were excluded from further analyses. Quality checks of the assemblies were performed using QUAST version 4.6 [71]. Genome assemblies were annotated using Prokka version 1.13 [72]. Individual Genbank/SRA accession numbers for FASTQ reads, genome assemblies, and source are given in Table S1.

### 4.5. Identification of Botulinum Neurotoxin Subtypes and Accessory Protein Configuration

The Prokka-annotated features were searched with representatives of the A, B, C, D, E, F, G, FA, X and Ebont/J toxins [9,16] using BLAST in the BioEdit version 7.2.5 program [73]). Incomplete, fragmented and inactivated toxins were manually generated from the individual genome sequences. Toxins were assigned to specific types after alignment of the toxin amino acid sequences with the reference types [16] using the Muscle module of MEGA7 [59], and subsequent generation of a Neighbour-Joining tree and clustering with reference toxins. Accessory genes were identified using BLAST, using reference sequences for the *ha* and *orfX* configurations [3].

### 4.6. Pangenome Analyses, Target Gene Identification and in Silico PCR

The *C. botulinum* Group II pangenome was identified using Roary version 3.12 [67], using the Prokka-annotated assemblies, with 80% and 90% BLAST cut-off percentages, and paralog clustering both switched off and on [74]. The Roary outputs were subsequently analysed using Scoary [75], with the no-pairwise option to speed up analysis, and a Bonferroni corrected *p*-value cut-off of 0.05. Gene distribution was studied and only genes present in ≥90% in the tester group and less than ≤10% of the comparator group were further studied. The plots for the pangenome distribution were generated with roary_plots (https://github.com/sanger-pathogens/Roary/tree/master/contrib/roary_plots). In silico PCR was performed using MIST [76].

### 4.7. Core Genome SNPs Analysis and Phylogenetic Trees

Core genome SNPs were identified using the ParSNP program [63] with the “-x -a 13” settings [77]. Treegraph v2 [64], MEGA7 [59] and Figtree (http://tree.bio.ed.ac.uk/software/figtree/) were used to annotate and visualize the phylogenetic trees.

## Supplementary Materials

The following are available online at https://www.mdpi.com/2072-6651/12/5/306/s1, Table S1, Strains and genome sequences of *C. botulinum* Group II used in this study; Table S2, Strain ID Figure 2, Figure 3, Figure 4; Table S3, Strain ID Figure 5; Figure S1, Alignment of the reference subtype E1 botulinum toxin.
