# Supplementary material for: Pan-Genomic Analysis of Clostridium botulinum Group II (Non-Proteolytic C. botulinum) Associated with Foodborne Botulism and Isolated from the Environment
Source: Toxins (Basel). 2020 May 8;12(5):306. doi: 10.3390/toxins12050306 (PMC7291236; doi:10.3390/toxins12050306)
Supplement: Supplementary file 1 [file toxins-12-00306-s001.zip › toxins-766361 sp/Supplementary_figure1 5.5.2020.pdf]

Toxins **2020**, *12*, 306; doi:10.3390/toxins12050306

Supplementary Materials: Pan-genomic Analysis of *Clostridium botulinum* Group II (Non-proteolytic *C. botulinum*) Associated with Foodborne Botulism and Isolated from the Environment

Jason Brunt, Arnoud H. M. van Vliet, Sandra C. Stringer, Andrew T. Carter, Miia Lindström and Michael W. Peck

A

CB0370

```
/region_name="Peptidase_M27", Clostridial neurotoxin zinc protease
/region_name="Toxin_trans", Clostridium neurotoxin, Translocation domain
/region_name="Toxin_R_bind_N", Clostridium neurotoxin, N-terminal receptor binding
/region_name="Toxin_R_bind_C", Clostridium neurotoxin, C-terminal receptor binding

ToxE1_reference          MPKINSFNYNDPVNDRTILYIKPGGCQEFYKSFNIMKNIWIIPERNVIGT
ToxE1_CB0370             MPKINSFNYNDPVNDRTILYIKPGGCQEFYKSFNIMKNIWIIPERNVIGT
                         **************************************************

ToxE1_reference          TPQDFHPPTSLKNGDSSYYDPNYLQSDEEKDRFLKIVTKIFNRINNNLSG
ToxE1_CB0370             TPQDFHPPTSLKNGDSSYYDPNYLQSDEEKDRFLKIVTKIFNRINNNLSG
                         **************************************************

ToxE1_reference          GILLEELSKANPYLGNDNTPDNQFHIGDASAVEIKFSNGSQDILLPNVII
ToxE1_CB0370             GILLEELSKANPYLGNDNTPDNQFHIGDASAVEIKFSNGSQDILLPNVII
                         **************************************************

ToxE1_reference          MGAEPDLFETNSSNISLRNNYMPSNHGFGSIAIVTFSPEYSFRFNDNSMN
ToxE1_CB0370             MGAEPDLFETNSSNISLRNNYMPSNHGFGSIAIVTFSPEYSFRFNDNSMN
                         **************************************************

ToxE1_reference          EFIQDPALTLMHELIHSLHGLYGAKGITTKYTITQKQNPLITNIRGTNIE
ToxE1_CB0370             EFIQDPALTLMHELIHSLHGLYGAKGITTKYTITQKQNPLITNIRGTNIE
                         **************************************************

ToxE1_reference          EFLTFGGTDLNIITSAQSNDIYTNLLADYKKIASKLSKVQVSNPLLNPYK
ToxE1_CB0370             EFLTFGGTDLNIITSAQSNDIYTNLLADYKKIASKLSKVQVSNPLLNPYK
                         **************************************************

ToxE1_reference          DVFEAKYGLDKDASGIYSVNINKFNDIFKKLYSFTEFDLATKFQVKCRQT
ToxE1_CB0370             DVFEAKYGLDKDASGIYSVNINKFNDIFKKLYSFTEFDLATKFQVKCRQT
                         **************************************************

ToxE1_reference          YIGQYKYFKLSNLLNDSIYNISEGYNINNLKVNFRGQNANLNPRIITPIT
ToxE1_CB0370             YIGQYKYFKLSNLLNDSIYNISEGYNINNLKVNFRGQNANLNPRIITPIT
                         **************************************************

ToxE1_reference          GRGLVKKIIRFCKNIVSVKGIRKSICIEINNGELFFVASENSYNDDNINT
ToxE1_CB0370             GRGLVKKIIRFCKNIVSVKGIRKSICIEINNGELFFVASENSYNDDNINT
                         **************************************************

ToxE1_reference          PKEIDDTVTSNNNYENDLDQVILNFNSESAPGLSDEKLNLTIQNDAYIPK
ToxE1_CB0370             PKEIDDTVTSNNNYENDLDQVILNFNSESAPGLSDEKLNLTIQNDAYIPK
                         **************************************************

ToxE1_reference          YDSNGTSDIEQHDVNELNVFFYLDAQKVPEGENNVNLTSSIDTALLEQPK
ToxE1_CB0370             YDSNGTSDIEQHDVNELNVFFYLDAQKVPEGENNVNLTSSIDTALLEQPK
                         **************************************************

ToxE1_reference          IYTFFSSEFINNVNKPVQAALFVSWIQQVLVDFTTEANQKSTVDKIADIS
ToxE1_CB0370             IYTFFSSEFINNVNKPVQAALFVGWIQQVLVDFTTEANQKSTVDKIADIS
                         ***********************.**************************
```

```
ToxE1_reference          IVVPYIGLALNIGNEAQKGNFKDALELLGAGILLEFEPELLIPTILVFTI
ToxE1_CB0370             IVVPYIGLALNIGNEAQKGNFKDALELLGAGILLEFEPELLIPTILVFTI
                         **************************************************

ToxE1_reference          KSFLGSSDNKNKVIKAINNALKERDEKWKEVYSFIVSNWMTKINTQFNKR
ToxE1_CB0370             KSFLGSSDNKNKVIKAINNALKERDEKWKEVYSFIVSNWMTKINTQFNKR
                         **************************************************

ToxE1_reference          KEQMYQALQNQVNAIKTIIESKYNSYTLEEKNELTNKYDIKQIENELNQK
ToxE1_CB0370             KEQMYQALQNQVNALKTIIESKYNSYTLEEKNELTNKYNIEQIENELNQK
                         **************:***********************:*:*********

ToxE1_reference          VSIAMNNIDRFLTESSISYLMKLINEVKINKLREYDENVKTYLLNYIIQH
ToxE1_CB0370             VSIAMNNIEIFLTESSISYLMKLINEVKINKLREYDENVKTYLLDYIIKH
                         ********: **********************************:***:*

ToxE1_reference          GSILGESQQELNSMVTDTLNNSIPFKLSSYTDDKILISYFNKFFKRIKSS
ToxE1_CB0370             GSILGESQQELNSMVIDTLNNSIPFKLSSYTDDKILISYFNKFFKRIKSS
                         *************** **********************************

ToxE1_reference          SVLNMRYKNDKYVDTSGYDSNININGDVYKYPTNKNQFGIYNDKLSEVNI
ToxE1_CB0370             SVLNMRYKNDKYVDTSGYDSNININGDVYKYPTNKNQFGIYNDKLSEVNI
                         **************************************************

ToxE1_reference          SQNDYIIYDNKYKNFSISFWVRIPNYDNKIVNVNNEYTIINCMRDNNSGW
ToxE1_CB0370             SQNDYIIYDNKYKNFSISFWVRIPNYDNKIVNVNNEYTIINCMRDNNSGW
                         **************************************************

ToxE1_reference          KVSLNHNEIIWTLQDNAGINQKLAFNYGNANGISDYINKWIFVTITNDRL
ToxE1_CB0370             KVSLNHNEIIWTLQDNAGINQKLAFNYGNANGISDYINKWIFVTITNDRL
                         **************************************************

ToxE1_reference          GDSKLYINGNLIDQKSILNLGNIHVSDNILFKIVNCSYTRYIGIRYFNIF
ToxE1_CB0370             GDSKLYINGNLIDQKSILNLGNIHVSDNILFKIVNCSYTRYIAIRYFNIF
                         ******************************************.*******

ToxE1_reference          DKELDETEIQTLYSNEPNTNILKDFWGNYLLYDKEYYLLNVLKPNNFIDR
ToxE1_CB0370             DKELDETEIQTLYSNEPNTNILKDFWGNYLLYDKEYYLLNVLKPNNFIDR
                         **************************************************

ToxE1_reference          RKDSTLSINNIRSTILLANRLYSGIKVKIQRVNNSSTNDNLVRKNDQVYI
ToxE1_CB0370             KKDSTLSINNIRSTILLANRLYSGIKVKIQRVNNSSTNDNLVRKNDQVYI
                         :*************************************************

ToxE1_reference          NFVASKTHLFPLYADTATTNKEKTIKISSSGNRFNQVVVMNSVGNNCTMN
ToxE1_CB0370             NFVASKTHLFPLYADTATTNKEKTIKISSSGNRFNQVVVMNSVGNNCTMN
                         **************************************************

ToxE1_reference          FKNNNGNNIGLLGFKADTVVASTWYYTHMRDHTNSNGCFWNFISEEHGWQ
ToxE1_CB0370             FKNNNGNNIGLLGFKADTVVASTWYYTHMRDNTNSNGCFWNFISEEHGWQ
                         *******************************:******************

ToxE1_reference          EK
ToxE1_CB0370             EK
                         **
```

Peptidase_M27 domain: no differences
Translocation domain: 7 conservative substitutions, 2 other changes
N-terminal receptor binding: 1 conservative substitution (G1043A)
C-terminal receptor binding: 2 conservative substitutions (R1101K, H1232N)

B

CB0355

```
/region_name="Peptidase_M27", Clostridial neurotoxin zinc protease
/region_name="Toxin_trans", Clostridium neurotoxin, Translocation domain
/region_name="Toxin_R_bind_N", Clostridium neurotoxin, N-terminal receptor binding
/region_name="Toxin_R_bind_C", Clostridium neurotoxin, C-terminal receptor binding

ToxE1_reference          MPKINSFNYNDPVNDRTILYIKPGGCQEFYKSFNIMKNIWIIPERNVIGT
ToxE1_CB0355             MPKINSFNYNDPVNDRTILYIKPGGCQEFYKSFNIMKNIWIIPERNVIGT
                         **************************************************

ToxE1_reference          TPQDFHPPTSLKNGDSSYYDPNYLQSDEEKDRFLKIVTKIFNRINNNLSG
ToxE1_CB0355             TPQDFHPPTSLKNGDSSYYDPNYLQSDEEKDRFLKIVTKIFNRINNNLSG
                         **************************************************

ToxE1_reference          GILLEELSKANPYLGNDNTPDNQFHIGDASAVEIKFSNGSQDILLPNVII
ToxE1_CB0355             GILLEELSKANPYLGNDNTPDNQFHIGDASAVEIKFSNGSQDILLPNVII
                         **************************************************

ToxE1_reference          MGAEPDLFETNSSNISLRNNYMPSNHGFGSIAIVTFSPEYSFRFNDNSMN
ToxE1_CB0355             MGAEPDLFETNSSNISLRNNYMPSNHGFGSIAIVTFSPEYSFRFNDNSMN
                         **************************************************

ToxE1_reference          EFIQDPALTLMHELIHSLHGLYGAKGITTKYTITQKQNPLITNIRGTNIE
ToxE1_CB0355             EFIQDPALTLMHELIHSLHGLYGAKGITTKYTITQKQNPLITNIRGTNIE
                         **************************************************

ToxE1_reference          EFLTFGGTDLNIITSAQSNDIYTNLLADYKKIASKLSKVQVSNPLLNPYK
ToxE1_CB0355             EFLTFGGTDLNIITSAQSNDIYTNLLADYKKIASKLSKVQVSNPLLNPYK
                         **************************************************

ToxE1_reference          DVFEAKYGLDKDASGIYSVNINKFNDIFKKLYSFTEFDLATKFQVKCRQT
ToxE1_CB0355             DVFEAKYGLDKDASGIYSVNINKFNDIFKKLYSFTEFDLATKFQVKCRQT
                         **************************************************

ToxE1_reference          YIGQYKYFKLSNLLNDSIYNISEGYNINNLKVNFRGQNANLNPRIITPIT
ToxE1_CB0355             YIGQYKYFKLSNLLNDSIYNISEGYNINNLKVNFRGQNANLNPRIITPIT
                         **************************************************

ToxE1_reference          GRGLVKKIIRFCKNIVSVKGIRKSICIEINNGELFFVASENSYNDDNINT
ToxE1_CB0355             GRGLVKKIIRFCKNIVSVKGIRKSICIEINNGELFFVASENSYNDDNINT
                         **************************************************

ToxE1_reference          PKEIDDTVTSNNNYENDLDQVILNFNSESAPGLSDEKLNLTIQNDAYIPK
ToxE1_CB0355             PKEIDDTVTSNNNYENDLDQVILNFNSESAPGLSDEKLNLTIQNDAYIPK
                         **************************************************

ToxE1_reference          YDSNGTSDIEQHDVNELNVFFYLDAQKVPEGENNVNLTSSIDTALLEQPK
ToxE1_CB0355             YDSNGTSDIEQHDVNELNVFFYLDAQKVPEGENNVNLTSSIDTALLEQPK
                         **************************************************

ToxE1_reference          IYTFFSSEFINNVNKPVQAALFVSWIQQVLVDFTTEANQKSTVDKIADIS
ToxE1_CB0355             IYTFFSSEFINNVNKPVQAALFVGWIQQVLVDFTTEANQKSTVDKIADIS
                         ***********************.**************************

ToxE1_reference          IVVPYIGLALNIGNEAQKGNFKDALELLGAGILLEFEPELLIPTILVFTI
ToxE1_CB0355             IVVPYIGLALNIGNEAQKGNFKDALELLGAGILLEFVPELLIPTILVFTI
                         ************************************ *************

ToxE1_reference          KSFLGSSDNKNKVIKAINNALKERDEKWKEVYSFIVSNWMTKINTQFNKR
ToxE1_CB0355             KSFLGSSDNKNKVIKAINNALKERDEKWKEVYSFIVSNWITKINTQFNKR
                         ***************************************:**********

ToxE1_reference          KEQMYQALQNQVNAIKTIIESKYNSYTLEEKNELTNKYDIKQIENELNQK
ToxE1_CB0355             KEQMYQALQNQVNALKTIIEFKYNSYTLEEKKELKNNYDIEQIENELNQK
                         **************:***** **********:**.*:***:*********
```

```
ToxE1_reference          VSIAMNNIDRFLTESSISYLMKLINEVKINKLREYDENVKTYLLNYIIQH
ToxE1_CB0355             VSIAMNNIEIFLTESSISYLMKLINEVKINKLREYDENVKTYLLDYIIKH
                         ********: **********************************:***:*

ToxE1_reference          GSILGESQQELNSMVTDTLNNSIPFKLSSYTDDKILISYFNKFFKRIKSS
ToxE1_CB0355             GSILGESQQELNSMVIDTLNNSIPFKLSSYTDDKILISYFNKFFKRIKSS
                         *************** **********************************

ToxE1_reference          SVLNMRYKNDKYVDTSGYDSNININGDVYKYPTNKNQFGIYNDKLSEVNI
ToxE1_CB0355             SVLNMRYKNDKYVDTSGYDSNININGDVYKYPTNKNQFGIYNDKLSEVNI
                         **************************************************

ToxE1_reference          SQNDYIIYDNKYKNFSISFWVRIPNYDNKIVNVNNEYTIINCMRDNNSGW
ToxE1_CB0355             SQNDYIIYDNKYKNFSISFWVRIPNYDNKIVNVNNEYTIINCMRDNNSGW
                         **************************************************

ToxE1_reference          KVSLNHNEIIWTLQDNAGINQKLAFNYGNANGISDYINKWIFVTITNDRL
ToxE1_CB0355             KVSLNHNEIIWTLQDNAGINQKLAFNYGNANGISDYINKWIFVTITNDRL
                         **************************************************

ToxE1_reference          GDSKLYINGNLIDQKSILNLGNIHVSDNILFKIVNCSYTRYIGIRYFNIF
ToxE1_CB0355             GDSKLYINGNLIDQKSILNLGNIHVSDNILFKIVNCSYTRYIAIRYFNIF
                         ******************************************.*******

ToxE1_reference          DKELDETEIQTLYSNEPNTNILKDFWGNYLLYDKEYYLLNVLKPNNFIDR
ToxE1_CB0355             DKELDETEIQTLYSNEPNTNILKDFWGNYLLYDKEYYLLNVLKPNNFIDR
                         **************************************************

ToxE1_reference          RKDSTLSINNIRSTILLANRLYSGIKVKIQRVNNSSTNDNLVRKNDQVYI
ToxE1_CB0355             KKDSTLSINNIRSTILLANRLYSGIKVKIQRVNNSSTNDNLVRKNDQVYI
                         :*************************************************

ToxE1_reference          NFVASKTHLFPLYADTATTNKEKTIKISSSGNRFNQVVVMNSVGNNCTMN
ToxE1_CB0355             NFVASKTHLFPLYADTATTNKEKTIKISSSGNRFNQVVVMNSVGNNCTMN
                         **************************************************

ToxE1_reference          FKNNNGNNIGLLGFKADTVVASTWYYTHMRDHTNSNGCFWNFISEEHGWQ
ToxE1_CB0355             FKNNNGNNIGLLGFKADTVVASTWYYTHMRDNTNSNGCFWNFISEEHGWQ
                         *******************************:******************

ToxE1_reference          EK
ToxE1_CB0355             EK
                         **

Peptidase_M27 domain: no differences
Translocation domain: 10 conservative substitutions, 4 other changes
N-terminal receptor binding: 1 conservative substitution (G1043A)
C-terminal receptor binding: 2 conservative substitutions (R1101K, H1232N)
```

Figure S1. Alignment of the reference subtype E1 botulinum toxin (from strain Beluga (CB1085), labeled ToxE1_reference) with **A**) the subtype E1 toxin encoded by isolate CB0370 (*C. botulinum* 1304E, labeled ToxE1_CB0370) and **B**) the subtype E1 toxin encoded by isolate CB0355 (strain SAR, labeled ToxE1_CB0355, and the identical toxins from strains Saroma, IFR 18/112, IFR 18/132). Asterisk symbols represent identical amino acids, colon and full stop symbols represent conservative substitutions. Differences between the reference subtype E1 toxin and the CB0370/CB0355 toxin are highlighted in red. Predicted domains of the toxin are indicated in yellow, green, grey and light blue, with relevant mutations indicated at the end of the alignment.
